# Scope of Algae as Third Generation Biofuels

**DOI:** 10.3389/fbioe.2014.00090

**Published:** 2015-02-11

**Authors:** Shuvashish Behera, Richa Singh, Richa Arora, Nilesh Kumar Sharma, Madhulika Shukla, Sachin Kumar

**Affiliations:** ^1^Biochemical Conversion Division, Sardar Swaran Singh National Institute of Renewable Energy, Kapurthala, Punjab, India

**Keywords:** algae, microalgae, biofuels, bioethanol, biogas, biodiesel, biohydrogen

## Abstract

An initiative has been taken to develop different solid, liquid, and gaseous biofuels as the alternative energy resources. The current research and technology based on the third generation biofuels derived from algal biomass have been considered as the best alternative bioresource that avoids the disadvantages of first and second generation biofuels. Algal biomass has been investigated for the implementation of economic conversion processes producing different biofuels such as biodiesel, bioethanol, biogas, biohydrogen, and other valuable co-products. In the present review, the recent findings and advance developments in algal biomass for improved biofuel production have been explored. This review discusses about the importance of the algal cell contents, various strategies for product formation through various conversion technologies, and its future scope as an energy security.

## Introduction

The requirement of energy for the mankind is increasing day by day. The major source of energy is based on fossil fuels only. Thus, the scarcity of fossil fuels, rising price of petroleum based fuels, energy protection, and increased global warming resulted in focusing on renewable energy sources such as solar, wind, hydro, tidal, and biomass worldwide (Goldemberg and Guardabassi, 2009; Dragone et al., 2010; Rajkumar et al., 2014).

Different biomass from various sources like agricultural, forestry, and aquatic have been taken into consideration as the feedstocks for the production of several biofuels such as biodiesel (Boyce et al., 2008; Yanqun et al., 2008), bioethanol (Behera et al., 2014), biohydrogen (Marques et al., 2011), bio-oil (Shuping et al., 2010), and biogas (Hughes et al., 2012; Singh et al., 2014). However, the environmental impact raised from burning of fuels has a great impact on carbon cycle (carbon balance), which is related to the combustion of fossil fuels. Besides, exhaustion of different existing biomass without appropriate compensation resulted in huge biomass scarcity, emerging environmental problems such as deforestation and loss of biodiversity (Goldemberg, 2007; Li et al., 2008; Saqib et al., 2013).

Recently, researchers and entrepreneurs have focused their interest, especially on the algal biomass as the alternative feedstock for the production of biofuels. Moreover, algal biomass has no competition with agricultural food and feed production (Demirbas, 2007). The photosynthetic microorganisms like microalgae require mainly light, carbon dioxide, and some nutrients (nitrogen, phosphorus, and potassium) for its growth, and to produce large amount of lipids and carbohydrates, which can be further processed into different biofuels and other valuable co-products (Brennan and Owende, 2010; Nigam and Singh, 2011). Interestingly, the low content of hemicelluloses and about zero content of lignin in algal biomass results in an increased hydrolysis and/or fermentation efficiency (Saqib et al., 2013). Other than biofuels, algae have applications in human nutrition, animal feed, pollution control, biofertilizer, and waste water treatment (Thomas, 2002; Tamer et al., 2006; Crutzen et al., 2007; Hsueh et al., 2007; Choi et al., 2012). Therefore, the aim of the current review is to explore the scope of algae for the production of different biofuels and evaluation of its potential as an alternative feedstock.

## Algae: Source of Biofuels

Generally, algae are a diverse group of prokaryotic and eukaryotic organisms ranging from unicellular genera such as *Chlorella* and diatoms to multicellular forms such as the giant kelp, a large brown alga that may grow up to 50 m in length (Li et al., 2008). Algae can either be autotrophic or heterotrophic. The autotrophic algae require only inorganic compounds such as CO_2_, salts, and a light energy source for their growth, while the heterotrophs are non-photosynthetic, which require an external source of organic compounds as well as nutrients as energy sources (Brennan and Owende, 2010). Microalgae are very small in sizes usually measured in micrometers, which normally grow in water bodies or ponds. Microalgae contain more lipids than macroalgae and have the faster growth in nature (Lee et al., 2014a). There are about more than 50,000 microalgal species out of which only about 30,000 species have been taken for the research study (Surendhiran and Vijay, 2012; Richmond and Qiang, 2013; Rajkumar et al., 2014). The short harvesting cycle of algae is the key advantage for its importance, which is better than other conventional crops having harvesting cycle of once or twice in a year (Chisti, 2007; Schenk et al., 2008). Therefore, the main focus has been carried out on algal biomass for its application in biofuel area.

There are several advantages of algal biomass for biofuels production: (a) ability to grow throughout the year, therefore, algal oil productivity is higher in comparison to the conventional oil seed crops; (b) higher tolerance to high carbon dioxide content; (c) the consumption rate of water is very less in algae cultivation; (d) no requirement of herbicides or pesticides in algal cultivation; (e) the growth potential of algal species is very high in comparison to others; (f) different sources of wastewater containing nutrients like nitrogen and phosphorus can be utilized for algal cultivation apart from providing any additional nutrient; and (g) the ability to grow under harsh conditions like saline, brackish water, coastal seawater, which does not affect any conventional agriculture (Spolaore et al., 2006; Dismukes et al., 2008; Dragone et al., 2010). However, there are several disadvantages of algal biomass as feedstock such as the higher cultivation cost as compared to conventional crops. Similarly, harvesting of algae require high energy input, which is approximately about 20–30% of the total cost of production. Several techniques such as centrifugation, flocculation, floatation, sedimentation, and filtration are usually used for harvesting and concentrating the algal biomass (Demirbas, 2010; Ho et al., 2011).

**Figure 1:**
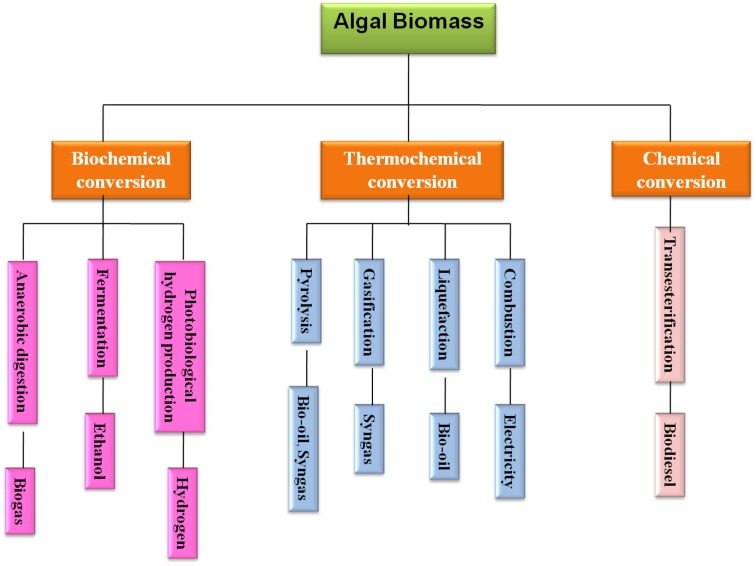
**Algal biomass conversion process for biofuel production**.

The algae can be converted into various types of renewable biofuels including bioethanol, biodiesel, biogas, photobiologically produced biohydrogen, and further processing for bio-oil and syngas production through liquefaction and gasification, respectively (Kraan, 2013). The conversion technologies for utilizing algal biomass to energy sources can be categorized into three different ways, i.e., biochemical, chemical, and thermochemical conversion and make an algal biorefinery, which has been depicted in Figure 1. The biofuel products derived from algal biomass using these conversion routes have been explored in detail in the subsequent sections.

## Biodiesel Production

Biodiesel is a mixture of monoalkyl esters of long chain fatty acids [fatty acid methyl esters (FAME)], which can be obtained from different renewable lipid feedstocks and biomass. It can be directly used in different diesel engines (Clark and Deswarte, 2008; Demirbas, 2009). Studies to explore the microalgae as feedstock for the production of liquid fuels had been started for the mid-1980s. In order to solve the energy crisis, the extraction of lipids from diatoms was attempted by some German scientists during the period of World War-II (Cohen et al., 1995). The higher oil yield in algal biomass as compared to oil seed crops makes the possibility to convert into the biodiesel economically using different technologies. A comparative study between algal biomass and terrestrial plants for the production of biodiesel has been depicted in Table 1. The oil productivity (mass of oil produced per unit volume of the microalgal broth per day) depends on the algal growth rate and the biomass content of the species. The species of microalgae such as *Kirchneriella lunaris*, *Ankistrodesmus fusiformis*, *Chlamydocapsa bacillus*, and *Ankistrodesmus falcatus* with high levels of polyunsaturated FAME are generally preferred for the production of biodiesel (Nascimento et al., 2013). They commonly multiply their biomass with doubling time of 24 h during exponential growth. Oil content of microalgae is generally found to be very high, which exceed up to 80% by weight of its dry biomass. About 5,000–15,000 gal of biodiesel can be produced from algal biomass per acre per year, which reflects its potentiality (Spolaore et al., 2006; Chisti, 2007).

**Table 1:** **Comparative study between algal biomass and terrestrial plants for biodiesel production**.

Feedstock	Conditions	Biodiesel	Reference
**ALGAE**			
*Spirulina platensis*	Reaction temperature 55°C, 60% catalyst concentration, 1:4 algae biomass to methanol ratio, 450 rpm stirring intensity	60 g/kg lipid	Nautiyal et al. (2014)
*Nannochloropsis* sp.	Oil extraction with n-hexane, acidic transesterification	99 g/kg lipid	Susilaningsih et al. (2009)
*Scenedesmus* sp.	Alkaline (NaOH), temperature of 70°C	321.06 g/kg lipid	Kim et al. (2014)
	Acidic (H_2_SO_4_) catalyst, temperature of 70°C	282.23 g/kg lipid	
*Nannochloropsis salina*	Freeze drying of biomass, extraction with chloroform–methanol (1:1 ratio), alkali transesterification	180.78 g/kg lipid	Muthukumar et al. (2012)
*Chlorella marina*		100 g/kg lipid	
**TERRESTRIAL PLANTS**			
*Madhuca indica*	0.30–0.35 (v/v) methanol-to-oil ratio, 1% (v/v) H_2_SO_4_ as acid catalyst, 0.25 (v/v) methanol, 0.7% (w/v) KOH as alkaline catalyst	186.2 g/kg lipid	Ghadge and Raheman (2005)
*Pongamia pinnata*	Transesterification with methanol, NaOH as catalyst, temp. 60°C	253 g/kg lipid	Mamilla et al. (2011)
	Acid-catalyzed esterification by using 0.5% H_2_SO_4_, alkali-catalyzed transesterification	193.2 g/kg lipid	Naik et al. (2008)
*Azadirachta indica*	Reaction time of 60 min, 0.7% H_2_SO_4_ as acid catalyst, reaction temperature of 50°C, and methanol: oil ratio of 3:1	170 g/kg lipid	Awolu and Layokun (2013)
Soybean	Hydrotalcite as basic catalyst, methanol/oil molar ratio of 20:1, reaction time of 10 h	189.6 g/kg lipid	Martin et al. (2013)

However, there are some standards such as International Biodiesel Standard for Vehicles (EN14214) and American Society for Testing and Materials (ASTM), which are required to comply with the algal based biodiesel on the physical and chemical properties for its acceptance as substitute to fossil fuels (Brennan and Owende, 2010). The higher degree of polyunsaturated fatty acids of algal oils as compared to vegetable oils make susceptible for oxidation in the storage and further limits its utilization (Chisti, 2007). Some researchers have reported the different advantages of the algal biomass for the biodiesel production due to its high biomass growth and oil productivity in comparison to best oil crops (Chisti, 2007; Hossain et al., 2008; Hu et al., 2008; Rosenberg et al., 2008; Schenk et al., 2008; Rodolfi et al., 2009; Mutanda et al., 2011).

Algal biodiesel production involves biomass harvesting, drying, oil extraction, and further transesterification of oil, which have been described as below.

### Harvesting and drying of algal biomass

Unicellular microalgae produce a cell wall containing lipids and fatty acids, which differ them from higher animals and plants. Harvesting of algal biomass and further drying is important prior to mechanical and solvent extraction for the recovery of oil. Macroalgae can be harvested using nets, which require less energy while microalgae can be harvested by some conventional processes, which include filtration (Rossignol et al., 1999) flocculation (Liu et al., 2013; Prochazkova et al., 2013), centrifugation (Heasman et al., 2008), foam fractionation (Csordas and Wang, 2004), sedimentation, froth floatation, and ultrasonic separation (Bosma et al., 2003). Selection of harvesting method depends on the type of algal species.

Drying is an important method to extend shelf-life of algal biomass before storage, which avoids post-harvest spoilage (Munir et al., 2013). Most of the efficient drying methods like spray-drying, drum-drying, freeze drying or lyophilization, and sun-drying have been applied on microalgal biomass (Leach et al., 1998; Richmond, 2004; Williams and Laurens, 2010). Sun-drying is not considered as a very effective method due to presence of high water content in the biomass (Mata et al., 2010). However, Prakash et al. (2007) used simple solar drying device and succeed in drying *Spirulina* and *Scenedesmus* having 90% of moisture content. Widjaja et al. (2009) showed the effectiveness of drying temperature during lipid extraction of algal biomass, which affects both concentration of triglycerides and lipid yield. Further, all these processes possess safety and health issues (Singh and Gu, 2010).

### Extraction of oil from algal biomass

Unicellular microalgae produce a cell wall containing lipids and fatty acids, which differ them from higher animals and plants. In the literature, there are different methods of oil extraction from algae, such as mechanical and solvent extraction (Li et al., 2014). However, the extraction of lipids from microalgae is costly and energy intensive process.

#### Mechanical oil extraction

The oil from nuts and seeds is extracted mechanically using presses or expellers, which can also be used for microalgae. The algal biomass should be dried prior to this process. The cells are just broken down with a press to leach out the oil. About 75% of oil can be recovered through this method and no special skill is required (Munir et al., 2013). Topare et al. (2011) extracted oil through screw expeller by mechanical pressing (by piston) and osmotic shock method and recovered about 75% of oil from the algae. However, more extraction time is required as compared to other methods, which make the process unfavorable and less effective (Popoola and Yangomodou, 2006).

#### Solvent based oil extraction

Oil extraction using solvent usually recovers almost all the oil leaving only 0.5–0.7% residual oil in the biomass. Therefore, the solvent extraction method has been found to be suitable method rather than the mechanical extraction of oil and fats (Topare et al., 2011). Solvent extraction is another method of lipid extraction from microalgae, which involves two stage solvent extraction systems. The amount of lipid extracted from microalgal biomass and further yield of highest biodiesel depends mainly on the solvent used. Several organic solvents such as chloroform, hexane, cyclo-hexane, acetone, and benzene are used either solely or in mixed form (Afify et al., 2010). The solvent reacts on algal cells releasing oil, which is recovered from the aqueous medium. This occurs due to the nature of higher solubility of oil in organic solvents rather than water. Further, the oil can be separated from the solvent extract. The solvent can be recycled for next extraction. Out of different organic solvents, hexane is found to be most effective due to its low toxicity and cost (Rajvanshi and Sharma, 2012; Ryckebosch et al., 2012).

In case of using mixed solvents for oil extraction, a known quantity of the solvent mixture is used, for example, chloroform/methanol in the ratio 2:1 (v/v) for 20 min using a shaker and followed by the addition of mixture, i.e., chloroform/water in the ratio of 1:1 (v/v) for 10 min (Shalaby, 2011). Similarly, Pratoomyot et al. (2005) extracted oil from different algal species using the solvent system chloroform/methanol in the ratio of 2:1 (v/v) and found different fatty acid content. Ryckebosch et al. (2012) optimized an analytical procedure and found chloroform/methanol in the ratio 1:1 as the best solvent mixture for the extraction of total lipids. Similarly, Lee et al. (1998) extracted lipid from the green alga *Botryococcus braunii* using different solvent system and obtained the maximum lipid content with chloroform/methanol in the ratio of 2:1. Hossain et al., 2008 used hexane/ether in the ratio 1:1 (v/v) for oil extraction and allowed to settle for 24 h. Using a two-step process, Fajardo et al. (2007) reported about 80% of lipid recovery using ethanol and hexane in the two steps for the extraction and purification of lipids. Therefore, a selection of a most suitable solvent system is required for the maximum extraction of oil for an economically viable process.

Lee et al. (2009) compared the performance of various disruption methods, including autoclaving, bead-beating, microwaves, sonication, and using 10% NaCl solution in the extraction of *Botryococcus* sp., *Chlorella vulgaris*, and *Scenedesmus* sp, using a mixture of chloroform and methanol (1:1).

### Transesterification

**Figure 2:**
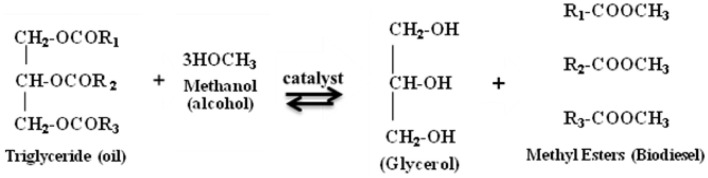
**Transesterification of oil to biodiesel**. *R*_1–3_ are hydrocarbon groups.

This is a process to convert algal oil to biodiesel, which involves multiple steps of reactions between triglycerides or fatty acids and alcohol. Different alcohols such as ethanol, butanol, methanol, propanol, and amyl alcohol can be used for this reaction. However, ethanol and methanol are used frequently for the commercial development due to its low cost and its physical and chemical advantages (Bisen et al., 2010; Surendhiran and Vijay, 2012). The reaction can be performed in the presence of an inorganic catalyst (acids and alkalies) or lipase enzyme. In this method, about 3 mol of alcohol are required for each mole of triglyceride to produce 3 mol of methyl esters (biodiesel) and 1 mol of glycerol (by-product) (Meher et al., 2006; Chisti, 2007; Sharma and Singh, 2009; Surendhiran and Vijay, 2012; Stergiou et al., 2013) (Figure 2). Glycerol is denser than biodiesel and can be periodically or continuously removed from the reactor in order to drive the equilibrium reaction. The presence of methanol, the co-solvent that keeps glycerol and soap suspended in the oil, is known to cause engine failure (Munir et al., 2013). Thus, the biodiesel is recovered by repeated washing with water to remove glycerol and methanol (Chisti, 2007).

The reaction rate is very slow by using the acid catalysts for the conversion of triglycerides to methyl esters, whereas the alkali-catalyzed transesterification reaction has been reported to be 4000 times faster than the acid-catalyzed reaction (Mazubert et al., 2013). Sodium and potassium hydroxides are the two commercial alkali catalysts used at a concentration of about 1% of oil. However, sodium methoxide has become the better catalyst rather than sodium hydroxide (Singh et al., 2006).

Kim et al. (2014) used *Scenedesmus* sp. for the biodiesel production through acid and alkali transesterification process. They reported 55.07 ± 2.18%, based on lipid by wt of biodiesel conversion using NaOH as an alkaline catalyst than using H_2_SO_4_ as 48.41 ± 0.21% of biodiesel production. In comparison to acid and alkalies, lipases as biocatalyst have different advantages as the catalysts due to its versatility, substrate selectivity, regioselectivity, enantioselectivity, and high catalytic activity at ambient temperature and pressure (Knezevic et al., 2004). It is not possible by some lipases to hydrolyze ester bonds at secondary positions, while some other group of enzymes hydrolyzes both primary and secondary esters. Another group of lipases exhibits fatty acids selectivity, and allow to cleave ester bonds at particular type of fatty acids. Luo et al. (2006) cloned the lipase gene lipB68 and expressed in *Escherichia coli* BL21 and further used it as a catalyst for biodiesel production. LipB68 could catalyze the transesterification reaction and produce biodiesel with a yield of 92% after 12 h, at a temperature of 20°C. The activity of the lipase enzyme with such a low temperature could provide substantial savings in energy consumption. However, it is rarely used due to its high cost (Sharma et al., 2001).

#### Extractive transesterification

It involves several steps to produce biodiesel such as drying, cell disruption, oils extraction, transesterification, and biodiesel refining (Hidalgo et al., 2013). The main problems are related with the high water content of the biomass (over 80%), which overall increases the cost of whole process.

#### *In situ* transesterification

This method skips the oil extraction step. The alcohol acts as an extraction solvent and an esterification reagent as well, which enhances the porosity of the cell membrane. Yields found are higher than via the conventional route, and waste is also reduced. Industrial biodiesel production involves release of extraction solvent, which contributes to the production of atmospheric smog and to global warming. Thus, simplification of the esterification processes can reduce the disadvantages of this attractive bio-based fuel. The single-step methods can be attractive solutions to reduce chemical and energy consumption in the overall biodiesel production process (Patil et al., 2012). A comparison of direct and extractive transesterification is given in Table 2.

**Table 2:** **Comparison of extractive transesterification and *in situ* methods (Haas and Wagner, 2011)**.

Sl. no.	Extractive transesterification	*In situ* transesterification
1	Low heating value	Heating value is high
2	Product yield is low	Higher product yield
3	Process is complex and time taking	Quick and simple operation process
4	Lipid loss during process	Avoided potential lipid loss
5	Waste water pollutes the environment	Reduced waste water pollutants
6	Production cost is high	Absence of harvesting and dewatering lowers the cost

## Bioethanol Production

**Figure 3:**
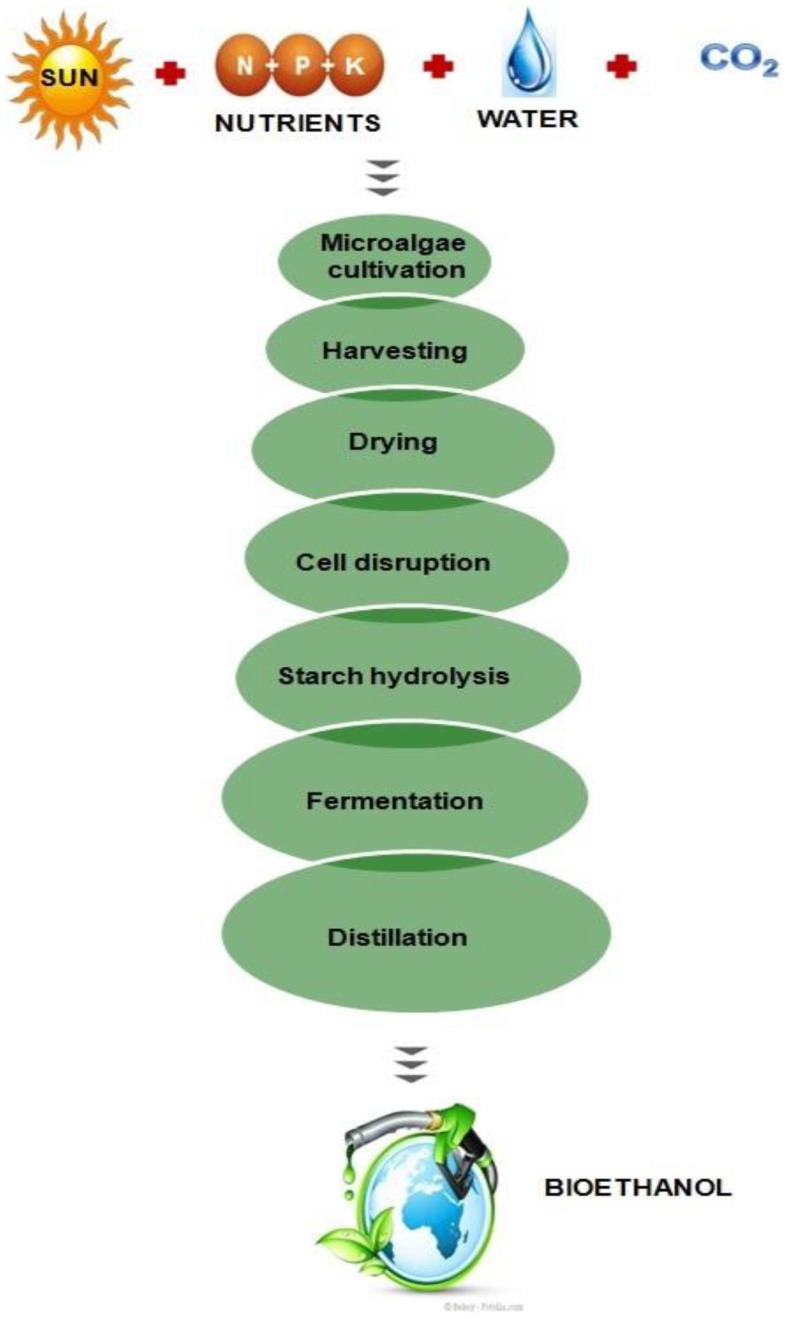
**Process for bioethanol production from microalgae**.

Several researchers have been reported bioethanol production from certain species of algae, which produce high levels of carbohydrates as reserve polymers. Owing to the presence of low lignin and hemicelluloses content in algae in comparison to lignocellulosic biomass, the algal biomass have been considered more suitable for the bioethanol production (Chen et al., 2013). Recently, attempts have been made (for the bioethanol production) through the fermentation process using algae as the feedstocks to make it as an alternative to conventional crops such as corn and soyabean (Singh et al., 2011; Nguyen and Vu, 2012; Chaudhary et al., 2014). A comparative study of algal biomass and terrestrial plants for the production of bioethanol has been given in Table 3. There are different micro and macroalgae such as *Chlorococcum* sp., *Prymnesium parvum*, *Gelidium amansii*, *Gracilaria* sp., *Laminaria* sp., *Sargassum* sp., and *Spirogyra* sp., which have been used for the bioethanol production (Eshaq et al., 2011; Rajkumar et al., 2014). These algae usually require light, nutrients, and carbon dioxide, to produce high levels of polysaccharides such as starch and cellulose. These polysaccharides can be extracted to fermentable sugars through hydrolysis and further fermentation to bioethanol and separated through distillation as shown in Figure 3.

**Table 3:** **Comparative study between algal biomass and terrestrial plants for bioethanol production**.

Feedstock	Conditions	Bioethanol	Reference
**ALGAE**			
*Chlorococcum infusionum*	Alkaline pre-treatment, temp. 120°C, *S. cerevisiae*	260 g ethanol/kg algae	Harun et al. (2011)
*Spirogyra*	Alkaline pre-treatment, synthetic media growth, saccharification of biomass by *Aspergillus niger*, fermentation by *S. cerevisiae*	80 g ethanol/kg algae	Eshaq et al. (2010)
*Chlorococcum humicola*	Acid pre-treatment, temp. 160°C, *S. cerevisiae*	520 g ethanol/kg microalgae	Harun and Danquah (2011a)
**TERRESTRIAL PLANTS**			
*Madhuca latifolia*	Strain *Zymomonas mobilis* MTCC 92, immobilized in *Luffa cylindrical* sponge disks, temp. 30°C	251.1 ± 0.012 g ethanol/kg flowers	Behera et al. (2011)
*Manihot esculenta*	Enzyme termamyl and amyloglucosidase, 1 N HCl, *Saccharomyces cerevisiae*, ca-alginate immobilization	189 ± 3.1 g ethanol/kg flour cassava	Behera et al. (2014)
Sugarcane bagasse	Acid (H_2_SO_4_) hydrolysis, *Kluyveromyces* sp. IIPE453, Fermentation at 50°C	165 g ethanol/kg bagasse	Kumar et al., 2014
Rice straw	Cellulase, β-glucosidase, solid state fermentation, strain *Trichoderma reesei* RUT C30, and *Aspergillus niger* MTCC 7956	93 g ethanol/kg pretreated rice straw	Sukumaran et al. (2008)

### Pre-treatment and saccharification

It has been reported that, the cell wall of some species of green algae like *Spirogyra* and *Chlorococcum* contain high level of polysaccharides. Microalgae such as *C. vulgaris* contains about 37% of starch on dry weight basis, which is the best source for bioethanol with 65% conversion efficiency (Eshaq et al., 2010; Lam and Lee, 2012). Such polysaccharide based biomass requires additional processing like pre-treatment and saccharification before fermentation (Harun et al., 2010). Saccharification and fermentation can also be carried out simultaneously using an amylase enzyme producing strain for the production of ethanol in a single step. Bioethanol from microalgae can be produced through the process, which is similar to the first generation technologies involving corn based feedstocks. However, there is limited literature available on the fermentation process of microalgae biomass for the production of bioethanol (Schenk et al., 2008; John et al., 2011).

The pre-treatment is an important process, which facilitates accessibility of biomass to enzymes to release the monosaccharides. Acid pre-treatment is widely used for the conversion of polymers present in the cell wall to simple forms. The energy consumption in the pre-treatment is very low and also it is an efficient process (Harun and Danquah, 2011a,b). Yazdani et al. (2011) found 7% (w/w) H_2_SO_4_ as the promising concentration for the pre-treatment of the brown macroalgae *Nizimuddinia zanardini* to obtain high yield of sugars without formation of any inhibitors. Candra and Sarinah (2011) studied the bioethanol production using red seaweed *Eucheuma cottonii* through acid hydrolysis. In this study, 5% H_2_SO_4_ concentration was used for 2 h at 100°C, which yielded 15.8 g/L of sugars. However, there are other alternatives to chemical hydrolysis such as enzymatic digestion and gamma radiation to make it more sustainable (Chen et al., 2012; Yoon et al., 2012; Schneider et al., 2013).

Similar to starch, there are certain polymers such as alginate, mannitol, and fucoidan present in the cell wall of various algae, which requires additional processing like pre-treatment and saccharification before fermentation. Another form of storage carbohydrate found in various brown seaweeds and microalgae is laminarin, which can be hydrolyzed by β-1,3-glucanases or laminarinases (Kumagai and Ojima, 2010). Laminarinases can be categorized into two groups such as exo- and endo-glucanases based on the mode of hydrolysis, which usually produces glucose and smaller oligosaccharides as the end product. Both the enzymes are necessary for the complete digestion of laminarin polymer (Lee et al., 2014b).

Markou et al. (2013) saccharified the biomass of *Spirulina* (*Arthrospira platensis*), fermented the hydrolyzate and obtained the maximum ethanol yield of 16.32 and 16.27% (g_ethanol_/g_biomass_) produced after pre-treatment with 0.5 N HNO_3_ and H_2_SO_4_, respectively. Yanagisawa et al. (2011) investigated the content of polysaccharide materials present in three types of seaweeds such as sea lettuce (*Ulva pertusa*), chigaiso (*Alaria crassifolia*), and agar weed (*Gelidium elegans*). These seaweeds contain no lignin, which is a positive signal for the hydrolysis of polysaccharides without any pre-treatment. Singh and Trivedi (2013) used *Spirogyra* biomass for the production of bioethanol using *Saccharomyces cerevisiae* and *Zymomonas mobilis*. In a method, they followed acid pre-treatment of algal biomass and further saccharified using α-amylase producing *Aspergillus niger*. In another method, they directly saccharified the biomass without any pre-treatment. The direct saccharification process resulted in 2% (w/w) more alcohol in comparison to pretreated and saccharified algal biomass. This study revealed that the pre-treatment with different chemicals are not required in case of *Spyrogyra*, which reflects its economic importance for the production of ethanol. Also, cellulase enzyme has been used for the saccharification of algal biomass containing cellulose. However, this enzyme system is more expensive than amylases and glucoamylases, and doses required for effective cellulose saccharification are usually very high. Trivedi et al. (2013) applied different cellulases on green alga *Ulva* for saccharification and found highest conversion efficiency of biomass into reducing sugars by using cellulase 22119 rather than viscozyme L, cellulase 22086 and 22128. In this experiment, they found a maximum yield of sugar 206.82 ± 14.96 mg/g with 2% (v/v) enzyme loading for 36 h at a temperature of 45°C.

### Fermentation

There are different groups of microorganisms like yeast, bacteria, and fungi, which can be used for the fermentation of the pretreated and saccharified algal biomass under anaerobic process for the production of bioethanol (Nguyen and Vu, 2012). Nowadays, *S. cerevisiae* and *Z. mobilis* have been considered as the bioethanol fermenting microorganisms. However, fermentation of mannitol, a polymer present in certain algae is not possible in anaerobic condition using these well known microorganisms and requires supply of oxygen during fermentation, which is possible only by *Zymobacter palmae* (Horn et al., 2000).

Certain marine red algae contain agar, a polymer of galactose and galactopyranose, which can be used for the production of bioethanol (Yoon et al., 2010). The biomass of red algae can be depolymerized into different monomeric sugars like glucose and galactose. In addition to mannitol and glucose, brown seaweeds contain about 14% of extra carbohydrates in the form of alginate (Wargacki et al., 2012). Horn et al. (2000) reported the presence of alginate, laminaran, mannitol, fucoidan, and cellulose in some brown seaweeds, which are good source of sugars. They fermented brown seaweed extract having mannitol using bacteria *Z. palmae* and obtained an ethanol yield of about 0.38 g ethanol/g mannitol.

In the literature, there are many advantages supporting microalgae as the promising substrate for bioethanol production. Hon-Nami (2006) used *Chlamydomonas perigranulata* algal culture and obtained different by-products such as ethanol and butanediol. Similarly, Yanagisawa et al. (2011) obtained glucose and galactose through the saccharification of agar weed (red seaweed) containing glucan and galactan and obtained 5.5% of ethanol concentration through fermentation using *S. cerevisiae* IAM 4178. Harun et al. (2010) obtained 60% more ethanol in case of lipid extracted microalgal biomass rather than intact algal biomass of *Chlorococcum* sp. This shows the importance of algal biomass for the production of both biodiesel and bioethanol.

## Biogas Production

Recently, biogas production from algae through anaerobic digestion has received a remarkable attention due to the presence of high polysaccharides (agar, alginate, carrageenan, laminaran, and mannitol) with zero lignin and low cellulose content. Mostly, seaweeds are considered as the excellent feedstock for the production of biogas. Several workers have demonstrated the fermentation of various species of algae like *Scenedesmus*, *Spirulina*, *Euglena*, and *Ulva* for biogas production (Samson and Leduy, 1986; Yen and Brune, 2007; Ras et al., 2011; Zhong et al., 2012; Saqib et al., 2013). The production of biogas using algal biomass in comparison to some terrestrial plants is shown in Table 4.

**Table 4:** **Comparative study between algal biomass and terrestrial plants for biogas production**.

Feedstock	Conditions	Biogas	Reference
**ALGAE**			
*Blue algae*	pH-6.8, microcystin (MC) biodegradation	189.89 mL/g of VS	Yuan et al. (2011)
*Chlamydomonas reinhardtii*	Drying as the pre-treatment, batch fermentation, temp. 38°C	587 mL/g of VS	Mussgnug et al. (2010)
*Scenedesmus obliquus*		287 mL/g of VS	
*Ulva* sp.	Batch reactor, Co-digestion with bovine slurry, temp. 35°C	191 mL/g of VS	Vanegas and Bartlett (2013)
*Laminaria digitata*		246 mL/g of VS	
*Saccorhiza polyschides*		255 mL/g of VS	
*Saccharina latissima*		235 mL/g of VS	
**TERRESTRIAL PLANTS**			
Banana stem	Pre-treatment: 6% NaOH in 55°C for 54 h. 37 ± 1°C for 40 days, batch	357.9 mL/g of VS	Zhang (2013)
Saline creeping wild ryegrass	35°C for 33 days, batch	251 mL/g of VS	Zheng (2009)
Rice straw	Pre-treatment: ammonia conc. 4% and moisture content 70%, temp. 35 ± 2°C, 65 days,120 rpm, batch	341.35 mL/g of VS	Yuan (2014)
Date palm tree wastes	Pre-treatment: alkaline, particle size 2–5 mm, temp. 40°C	342.2 mL/g of VS	Al-Juhaimi (2014)

Biogas is produced through the anaerobic transformation of organic matter present in the biodegradable feedstock such as marine algae, which releases certain gases like methane, carbon dioxide, and traces of hydrogen sulfide. The anaerobic conversion process involves basically four main steps. In the first step, the insoluble organic material and higher molecular mass compounds such as lipids, carbohydrates, and proteins are hydrolyzed into soluble organic material with the help of enzyme released by some obligate anaerobes such as *Clostridia* and *Streptococci*. The second step is called as acidogenesis, which releases volatile fatty acids (VFAs) and alcohols through the conversion of soluble organics with the involvement of enzymes secreted by the acidogenic bacteria. Further, these VFAs and alcohols are converted into acetic acid and hydrogen using acetogenic bacteria through the process of acetogenesis, which finally metabolize to methane and carbon dioxide by the methanogens (Cantrell et al., 2008; Vergara-Fernandez et al., 2008; Brennan and Owende, 2010; Romagnoli et al., 2011).

Sangeetha et al. (2011) reported the anaerobic digestion of green alga *Chaetomorpha litorea* with generation of 80.5 L of biogas/kg of dry biomass under 299 psi pressure. Vergara-Fernandez et al. (2008) evaluated digestion of the marine algae *Macrocystis pyrifera* and *Durvillaea antarctica* marine algae in a two-phase anaerobic digestion system and reported similar biogas productions of 180.4 (±1.5) mL/g dry algae/day with a methane concentration around 65%. However, in case of algae blend, same methane content was observed with low biogas yield. Mussgnug et al. (2010) reported biogas production from some selected green algal species like *Chlamydomonas reinhardtii* and *Scenedesmus obliquus* and obtained 587 and 287 mL biogas/g of volatile solids, respectively. Further, there are few studies, which have been conducted with microalgae showing the effect of different pre-treatment like thermal, ultrasound, and microwave for the high production of biogas (Gonzalez-Fernandez et al., 2012a,b; Passos et al., 2013).

However, there are different factors, which limit the biogas production such as requirement of larger land area, infrastructure, and heat for the digesters (Collet et al., 2011; Jones and Mayfield, 2012). The proteins present in algal cells increases the ammonium production resulting in low carbon to nitrogen ratio, which affects biogas production through the inhibition of growth of anaerobic microorganisms. Also, anaerobic microorganisms are inhibited by the sodium ions. Therefore, it is recommended to use the salt tolerating microorganisms for the anaerobic digestion of algal biomass (Yen and Brune, 2007; Brennan and Owende, 2010; Jones and Mayfield, 2012).

## Biohydrogen Production

Recently, algal biohydrogen production has been considered to be a common commodity to be used as the gaseous fuels or electricity generation. Biohydrogen can be produced through different processes like biophotolysis and photo fermentation (Shaishav et al., 2013). Biohydrogen production using algal biomass is comparative to that of terrestrial plants (Table 5). Park et al. (2011) found *Gelidium amansii* (red alga) as the potential source of biomass for the production of biohydrogen through anaerobic fermentation. Nevertheless, they found 53.5 mL of H_2_ from 1 g of dry algae with a hydrogen production rate of 0.518 L H_2_/g VSS/day. The authors found an inhibitor, namely, 5-hydroxymethylfurfural (HMF) produced through the acid hydrolysis of *G. amansii* that decreases about 50% of hydrogen production due to the inhibition. Thus, optimization of the pre-treatment method is an important step to maximize biohydrogen production, which will be useful for the future direction (Park et al., 2011; Shi et al., 2011). Saleem et al. (2012) reduced the lag time for hydrogen production using microalgae *Chlamydomonas reinhardtii* by the use of optical fiber as an internal light source. In this study, the maximum rate of hydrogen production in the presence of exogenic glucose and optical fiber was reported to be 6 mL/L culture/h, which is higher than other reported values.

**Table 5:** **Comparative study between algal biomass and terrestrial plants for biohydrogen production**.

Feedstock	Conditions	Biohydrogen	Reference
**ALGAE**			
*Gelidium amansii*	Hydrolysis at 150°C	53.5 mL of H_2_/g of dry algae	Park et al. (2011)
*Laminaria japonica*	Mesophilic condition (35 ± 1°C), pH of 7.5, anaerobic sequencing batch reactor, hydraulic retention time (HRT) of 6 days	71.4 mL H_2_/g of dry algae	Shi et al. (2011)
**TERRESTRIAL PLANTS**			
Bagasse	Strain *Klebsiella oxytoca* HP1, temp. 37.5°C, pH-7	107.8 ± 7.5 mL H_2_/g bagasse	Wu et al. (2010)
Corn stalk	Temp. 55°C, pH-7.4	61.4 mL/g of cornstalk	Cheng and Liu (2011)
Pretreated wheat straw	Strain *Caldicellulosiruptor saccharolyticus*, Temp. 70°C, pH-7.2	44.7 mL/g of dry wheat straw	Ivanova et al. (2009)
Wheat straw	Acid pre-treatment, simultaneous saccharification and fermentation (SSF)	141 mL/g VS	Nasirian et al. (2011)

Some of microalgae like blue green algae have glycogen instead of starch in their cells. This is an exception, which involves oxidation of ferrodoxin by the hydrogenase enzyme activity for the production of hydrogen in anaerobic condition. However, another function of this enzyme is to be involved in the detachment of electrons. Therefore, different researchers have focused for the identification of these enzyme activities having interactions with ferrodoxin and the other metabolic functions for microalgal photobiohydrogen production. They are also involved with the change of these interactions genetically to enhance the biohydrogen production (Gavrilescu and Chisti, 2005; Hankamer et al., 2007; Wecker et al., 2011; Yacoby et al., 2011; Rajkumar et al., 2014).

## Bio-Oil and Syngas Production

Bio-oil is formed in the liquid phase from algal biomass in anaerobic condition at high temperature. The composition of bio-oil varies according to different feedstocks and processing conditions, which is called as pyrolysis (Iliopoulou et al., 2007; Yanqun et al., 2008). There are several parameters such as water, ash content, biomass composition, pyrolysis temperature, and vapor residence time, which affect the bio-oil productivity (Fahmi et al., 2008). However, due to the presence of water, oxygen content, unsaturated and phenolic moieties, crude bio-oil cannot be used as fuel. Therefore, certain treatments are required to improve its quality (Bae et al., 2011). Bio-oils can be processed for power generation with the help of external combustion through steam and organic rankine cycles, and stirling engines. However, power can also be generated through internal combustion using diesel and gas-turbine engines (Chiaramonti et al., 2007). In literature, there are limited studies on algae pyrolysis compared to lignocellulosic biomass. Although, high yields of bio-oil occur through fluidized-bed fast pyrolysis processes, there are several other pyrolysis modes, which have been introduced to overcome their inherent disadvantages of a high level of carrier gas flow and excessive energy inputs (Oyedun et al., 2012). Demirbas (2006) investigated suitability of the microalgal biomass for bio-oil production and found the superior quality than the wood. Porphy and Farid (2012) produced bio-oil from pyrolysis of algae (*Nannochloropsis* sp.) at 300°C after lipid extraction, which composed of 50 wt% acetone, 30 wt% methyl ethyl ketone, and 19 wt% aromatics such as pyrazine and pyrrole. Similarly, Choi et al. (2014) carried out pyrolysis study on a species of brown algae *Saccharina japonica* at a temperature of 450°C and obtained about 47% of bio-oil yield.

Gasification is usually performed at high temperatures (800–1000°C), which converts biomass into the combustible gas mixture through partial oxidation process, called syngas or producer gas. Syngas is a mixture of different gases like CO, CO_2_, CH_4_, H_2_, and N_2_, which can also be produced through normal gasification of woody biomass. In this process, biomass reacts with oxygen and water (steam) to generate syngas. It is a low calorific gas, which can be utilized in the gas turbines or used directly as fuel. Different variety of biomass feedstocks can be utilized for the production of energy through the gasification process, which is an added advantage (Carvalho et al., 2006; Prins et al., 2006; Lv et al., 2007).

## Conclusion and Future Perspectives

Recently, it is a challenge for finding different alternative resources, which can replace fossil fuels. Due to presence of several advantages in algal biofuels like low land requirement for biomass production and high oil content with high productivity, it has been considered as the best resource, which can replace the liquid petroleum fuel. However, one of its bottlenecks is the low biomass production, which is a barrier for industrial production. Also, another disadvantage includes harvesting of biomass, which possesses high energy inputs. For an economic process development in comparison to others, a cost-effective and energy efficient harvesting methods are required with low energy input. Producing low-cost microalgal biofuels requires better biomass harvesting methods, high biomass production with high oil productivity through genetic modification, which will be the future of algal biology. Therefore, use of the standard algal harvesting technique, biorefinery concept, advances in photobioreactor design and other downstream technologies will further reduce the cost of algal biofuel production, which will be a competitive resource in the near future.

## Conflict of Interest Statement

The authors declare that the research was conducted in the absence of any commercial or financial relationships that could be construed as a potential conflict of interest.
